# The convergent epidemiology of tuberculosis and human cytomegalovirus infection

**DOI:** 10.12688/f1000research.14184.2

**Published:** 2018-04-30

**Authors:** Frank Cobelens, Nico Nagelkerke, Helen Fletcher

**Affiliations:** 1 Department of Global Health and Amsterdam Institute for Global Health and Development, Academic Medical Center, Meibergdreef 9, AZ, Amsterdam, 1105, Netherlands; 2 Department of Medical Microbiology, University of Manitoba, Basic Medical Sciences Building, 745 Bannatyne Avenue, Winnipeg, MB , R3E 0J9, Canada; 3 TB Centre, London School of Hygiene & Tropical Medicine, Keppel Street , London , WC1E 7HT, UK

**Keywords:** Tuberculosis, latent tuberculosis infection, human cytomegalovirus, epidemiology, age pattern, risk factor

## Abstract

Although several factors are known to increase the risk of tuberculosis, the occurrence of tuberculosis disease in an infected individual is difficult to predict. We hypothesize that active human cytomegalovirus infection due to recent infection, reinfection or reactivation plays an epidemiologically relevant role in the aetiology of tuberculosis by precipitating the progression from latent tuberculosis infection to disease. The most compelling support for this hypothesis comes from the striking similarity in age-sex distribution between the two infections, important because the age-sex pattern of tuberculosis disease progression has not been convincingly explained. Cytomegalovirus infection and tuberculosis have other overlapping risk factors, including poor socio-economic status, solid organ transplantation and, possibly, sexual contact and whole blood transfusion. Although each of these overlaps could be explained by shared underlying risk factors, none of the epidemiological observations refute the hypothesis. If this interaction would play an epidemiologically important role, important opportunities would arise for novel approaches to controlling tuberculosis.

## Introduction

With 10.4 million new cases and 1.7 million deaths per year, tuberculosis (TB) remains a major global health problem ^1^. Only 5%–15% of individuals infected with *Mycobacterium tuberculosis* (Mtb) ever develop TB disease, and over 50% of these do so within two years after infection ^2^. Although risk factors for progression to TB disease have been identified ^3^, disease occurrence cannot be accurately predicted ^4^.

Recent data suggest that infection with human cytomegalovirus (HCMV) is a predictor of TB disease in infants. In a cohort study of South African infants, an HCMV-specific IFN-γ T-cell response was associated with a 2.2-fold increased risk of TB disease over a period of up to 3 years. A similar response to Epstein-Barr virus (EBV) showed no such associations ^5^. HCMV-positive and HCMV-negative infants had distinct immune pathways associated with TB disease. Although CD8+ T-cell activation was a distinguishing feature of HCMV-positive infants, the proposed immunological mechanism was impairment of the natural killer (NK) cell response. In African infants, HCMV infection induced profound CD8+ T-cell and NK cell differentiation and poor physical growth ^5– 7^.

The possibility that this association between HCMV and TB disease progression is causal, also holds in adults, and thus merits further study is dependent on its epidemiological plausibility. Only few published studies have investigated epidemiological associations between the two diseases ^8– 10^. Despite this paucity of direct evidence we argue that the epidemiology of TB and HCMV share important similarities that make HCMV infection a plausible candidate as a cause of TB disease progression.

## Viral triggers of tuberculosis disease

Various etiological frameworks for TB disease progression have been developed. One proposed by Comstock considers TB disease the result of two hits or causes, one of which is Mtb infection, and the other (still) unknown ^11^. In this framework, factors that strongly increase the risk of TB disease such as HIV infection or anti-tumour necrosis alpha therapy may act as a second hit but would not account for all or most TB cases.

Several factors have been identified that increase the risk of disease progression, such as low body-mass index ^12^, diabetes ^13^, tobacco smoking ^14^, and alcohol abuse ^15^. As their effects are modest another framework has emerged that these are *predisposing conditions* for disease progression while other, yet unidentified *precipitating events* are needed to trigger progression to active disease ^16^. Among the precipitating events suggested are viral infections, possibly through induction of Type I interferons (IFN). Elevated Type I IFN signalling is a hallmark of viral control, however, Type I IFN is also associated with susceptibility to bacterial infections, including Mtb ^17– 22^. The Type I IFN response is tightly regulated by prostaglandins and the balance between prostoglandins PGE2 and LXA4 can be manipulated by Mtb to drive Type I IFN mediated necrosis and promote mybobacterial dissemination ^19, 23^. Type I IFN-associated impairment of the immunity against Mtb has been shown for influenza A ^21^. A role for influenza A infection has also been suggested by epidemiological data. Notification of TB tends to peak in the months after winter when most respiratory viruses circulate ^24^, and TB mortality has shown increases during influenza epidemics ^25, 26^. However, careful analysis of seasonality data suggests that it is TB transmission rather than disease progression that is increased in winter ^27^, and increased TB mortality during influenza epidemics may reflect increased case fatality among TB patients due to secondary influenza rather than increased TB incidence.

As with many viral infections, Type I IFN can control HCMV replication ^28, 29^. HCMV has been suggested in three studies from Nigeria, Russia and Uganda that all found higher prevalence or levels of IgG HCMV antibodies in diagnosed TB patients compared to healthy controls and patients diagnosed with other diseases ^8– 10^.

## Human cytomegalovirus infection

HCMV, human herpesvirus 5, is a double-stranded DNA virus. After primary infection, usually through mucosal contact, HCMV remains dormant in the host’s myeloid tissues but can reactivate if immunity is compromised. Primary infection is often asymptomatic but can present as mononucleosis with fever, pharyngo-tonsillitis and lymphadenopathy. In congenitally infected infants HCMV may cause severe generalized infection with high case fatality and neurologic sequelae ^30^. Generalized infection also occurs in severely immunocompromised adults, usually through reactivation. During primary infection and reactivation virus is shed in the urine, saliva, breast milk, cervical fluid and semen ^31^. Common routes of transmission are from mother to child during delivery, between children and by sexual contact. Transmission through blood transfusion and solid organ transplantations also occurs. 

HCMV viruses show genomic diversity, in particular in genes coding for envelope glycoproteins, and polymorphisms in these genes have been used to genotype strains ^32, 33^. Both immunocompromised and immunocompetent individuals can be re-infected and harbour multiple HCMV strains ^34– 36^.

Primary HCMV infection is characterized by profound expansion of antigen specific CD8+ and CD4+ T cells and NK cell populations with specificity for HCMV ^37^. HCMV expanded NK cells can display inappropriate homing to tissue infected with other pathogens and lower IFN-γ secretion in response to pathogens ^38^. HCMV infection drives the expansion of CD94/NKG2C NK cells and these cells are important for control of viral replication ^39^. In HCMV positive infants who progressed to TB disease in the South African cohort there was lower expression of CD94 and NKG2C (KLRD1 and KLRC3) transcripts and lower frequency of NK cells ^5^. The NKG2C receptor is encoded by the *KLRC2* gene which is deleted in approximately 10% of individuals ^40^. The *KLRC2* gene deletion is associated with lower numbers of mature NK cells and increased risk of HIV infection and disease progression ^41, 42^, as well as with susceptibility to autoimmune conditions and cancer ^40^. Susceptibility to TB in this infant population may be due to loss of control of CMV infection due to *KLRC2* gene defects in some individuals.

HCMV has multiple immune evasion strategies ^37^, which may make the microenvironment around latently infected myeloid cells suppressive to T-cell function, potentially creating an environment permissive for mycobacterial growth ^43, 44^. This may be through effects of HCMV on the systemic immune response, but also through local effects. The lung is a reservoir of HCMV infection ^45, 46^ and frequently the site of viral reactivation ^47^, which drives inflammation and in mice may cause pulmonary fibrosis ^48^. It is therefore possible that active HCMV (re)infection or reactivation of latent HCMV could precipitate progression to TB disease.

## Epidemiological convergence

Both Mtb and HCMV infections are ubiquitous ^1, 49^, and during millions of years of co-evolution have become highly human host-specific ^50– 52^. An animal reservoir has been described for neither Mtb nor HCMV (several monkey and rodent species have their distinct CMV species), implying that their epidemiological patterns are entirely determined by transmission between, and carriage by, humans. 

We hypothesize that immunologically active HCMV infection, whether primary, reactivation or re-infection, acts as (depending on one’s preferred framework) second-hit or precipitating factor for progression of latent TB infection to TB disease at an epidemiologically relevant scale. We base this on two arguments: their striking similarity in age distribution, and the existence of congruent risk factors [ Box 1].

Box 1. **Approach to evidence gathering**

We systematically searched PubMed for the following combinations of keywords: tuberculosis and cytomegalovirus; cytomegalovirus and prevalence or seroprevalence; cytomegalovirus and age; cytomegalovirus and reinfection; cytomegalovirus and sexual; tuberculosis and sexual; tuberculosis and sexual transmitted infections or Chlamydia or gonorrhoea or human papillomavirus; tuberculosis and blood transfusion; cytomegalovirus and blood transfusion; tuberculosis and gastrectomy; cytomegalovirus and renal dialysis; tuberculosis and renal dialysis; tuberculosis and organ transplantation; cytomegalovirus and organ transplantation.

We in addition made use of an extensive review of the literature on age-sex distribution of tuberculosis incidence published by Nagelkerke (2012) ^53^.

### Age distribution

**Figure 1.**
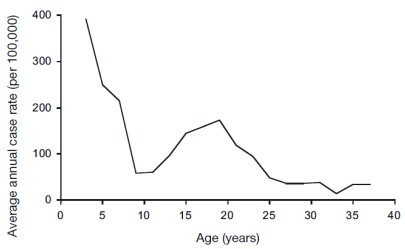
Age-specific incidence of tuberculosis disease among tuberculin-reactive children. Average annual rate of tuberculosis disease in a cohort of 82,269 Puerto Rican children with a positive tuberculin skin test, by age of disease occurrence. Children were enrolled in the period 1949–1951, and followed for 8 to 20 years. Figure reproduced with permission from Comstock *et al*. (1974) ^54^.

The probability of progressing from TB infection to disease has a highly typical age distribution. The classical description of this age pattern is by Comstock *et al*, who followed 82,269 Puerto Rican children reacting to tuberculin enrolled in 1949–1951 for 8 to 20 years [ Figure 1] ^54^. This pattern, confirmed in a systematic review of studies from the pre-chemotherapy era ^55^, is defined by a peak in the first 1–4 years of life, followed by a trough until early puberty, rising to a second peak around the age of 20 years. Analyses of notification and prevalence data from high-incidence countries show that incidence starts to rise again from the sixth decade ^56, 57^. Although several explanations for this age pattern have been suggested, none has been proven.

***Infants*.** Studies from the pre-chemotherapy era showed that, while the risk of infection with Mtb in the first year of life was over 10-fold lower than later in childhood, the risk of progression to disease once infected was much higher with up to 50% of infected infants developing disease ^55^. These high progression rates have been attributed to age-specific maturation of immune responses ^58^, although the mechanisms responsible for this vulnerability have not been elucidated ^59^.

HCMV infection in infants is common ^60^. Depending on the country and socio-economic status of the mother, between 10 and 60% of children are HCMV IgG seropositive (reflecting current or past active infection) by the age of 12–36 months ^61– 68^. Important causes are congenital infection and transmission through breastfeeding; >85% of HCMV seropositive women excrete virus in the breastmilk ^60, 69– 72^. Infants infected through breastfeeding do not develop disease, probably due to protection by maternal antibodies, but do shed virus in saliva and urine intermittently for months, by which they may transmit HCMV to other children and caregivers ^31, 60, 64, 73^. Shedding of HCMV shows a steep decline by the age of 5 years ^31^, coinciding with the age at which TB incidences drop ^54, 55^.

***Adolescents.*** The rate of progression to TB disease then remains low until puberty. Several studies have observed an increase in TB incidence from this age onward among children who were exposed to infectious TB patients or had a positive tuberculin response, leading to a peak in incidence in the first half of the third decade ^54, 74– 79^. This phenomenon has been attributed to hormonal changes, but again without a putative mechanistic pathway ^80^.

Most population-based studies of HCMV seroprevalence show exactly this age pattern: a slow increase in HCMV IgG seroprevalence up to the age of 10–15 years, followed by an acceleration during adolescence ^61, 63– 66, 68, 81– 88^. One explanation for this increase in seroprevalence is sexual transmission. Various studies found that HCMV conversion among women was associated with sexual activity ^89– 93^. However, as several studies of adolescents found no association of HCMV seroprevalence with sexual exposure ^83, 94, 95^, other transmission routes such as mouth-to-mouth kissing may also be important.

Another indication that HCMV infection may be implicated is the sex difference in TB disease progression in the second decade. For girls the increase in TB incidence starts 2–4 years earlier than for boys, and progression rates tend to remain higher in women than in men for the subsequent two decades, a pattern that was observed before the HIV era in various populations ^54, 75, 76, 78, 79, 96– 99^. This pattern is again reflected in that of HCMV infection. The acceleration of HCMV seroprevalence during puberty and adolescence is steeper in girls than in boys and is higher in women of childbearing age than in men in populations with relatively low HCMV seroprevalence ^49, 64, 65, 84, 100– 102^. Age-adjusted HCMV seroprevalence does not differ between men and women in populations with high seroprevalence ^49, 103^. This may be because IgG seroprevalence measures cumulative infection experience and thus ignores reinfection. HCMV reinfection, identified by DNA typing or strain-specific antibody responses, is a common occurrence in sexually exposed women ^104– 106^.

***Elderly*.** Although there is little data on TB progression rates in the elderly, age patterns of TB notifications suggest increased progression rates from the sixth decade onward ^1, 56^. In populations with declining incidence rates over the past decades this is partially a cohort effect, whereby younger generations have lower prevalence of latent infection ^107, 108^. However in high-incidence countries with little change in TB incidence, notification rates clearly increase at older age ^1^. This is also observed for TB prevalence in population surveys, suggesting that this is not explained by better access to diagnosis ^1^. HCMV infection has been implicated as a cause of age-related decrease in naïve T cells and increase in memory T cells known as immunosenescence ^109^. However, reactivation of HCMV infection is also common at old age, probably reflecting weakening immune control ^37^. Detection of viral DNA increases after the age of 60–70 years ^110, 111^, and viral DNA is frequently detected in urine and plasma of elderly people ^112, 113^.

### Congruent risk factors

Our hypothesis predicts that factors that drive CMV (re-)infection are also risk factors for TB. We highlight the four most important: socio-economic status, sexual contact, blood transfusion, and solid organ transplantation.

***Socio-economic status.*** Incidence and prevalence of CMV infection are associated with poor socio-economic status (SES), between countries as well as within countries and communities ^49, 64, 65, 100, 114– 116^. This includes association with crowding, in particular the number of young children in household ^117– 119^. Several studies found ethnicity or migrant status to be independently associated with age-adjusted CMV prevalence ^65, 84, 120^, which may partly reflect higher background infection rates in the country of origin. In a US study the association with ethnicity was explained by differences in exposure to infants and sexual risk ^93^.

Also the incidence of TB, often regarded as the archetypal poverty disease, shows a remarkable inverse gradient with SES at the household, regional and country level ^121– 123^. This association has been explained mainly by crowding in ill-ventilated spaces conducive to Mtb transmission ^124^, poor nutritional status ^12, 125^, alcohol abuse ^15^ and, possibly, indoor air pollution ^126^. Similarly, in low-incidence countries, TB incidences are higher in particular ethnic groups and immigrants ^127, 128^, which also may reflect socio-economic disparities and differences in background infection rates ^129^. Very few studies have attempted to investigate whether these and other known risk factors explain all of the observed variation in SES-related TB incidence ^130^.

***Sexual contact.*** The risk of CMV (re-)infection in adults is correlated with measures of sexual activity such as age at first intercourse, recent and lifetime number of sexual partners and condom use, as well as with prevalence of other sexually transmitted infections ^89, 90, 92, 131– 133^. Historically, TB has also been associated with sexual promiscuity in medical and popular literature (reviewed in 53) but no systematic epidemiological data exist. Investigation of associations between TB disease and sexually transmitted infections has been strongly dominated by HIV infection, which may obviously be a major confounder. There have been few studies from low HIV prevalence populations. One from China found an association between history of TB and human papilloma virus infection ^134^.

Interestingly, the declining TB mortality rates in The Netherlands and England and Wales in the 20 ^th^ century showed no surge during the Great Depression ^121, 135^, when SES status deteriorated thereby affecting several of these known risk factors, in particular nutritional status. They did however surge during and shortly after the Second World War ^121, 135^. In England and Wales this was not paralleled by major deterioration in nutritional status; in The Netherlands famine only started in the winter of 1944–45 while the increase in TB mortality started already from 1942 ^53^. In both countries during this period major increases were seen in sexually transmitted infections, mainly related to presence of large numbers of Allied and Axis troops ^53^.

***Blood transfusion.*** Transfusion-associated CMV infection occurs in particular following multiple transfusions of whole blood or granulocytes, and can be prevented by removal of white blood cells ^136– 138^. Increased incidences of TB have indeed been described in two categories of patients who in the past often received multiple whole blood transfusions: patients who underwent (partial) gastrectomy, mainly for bleeding gastric ulcers ^139, 140^, and patients with end-stage renal disease on haemodialysis ^141^. For both these categories alternative explanations for increased TB incidences are possible: low body mass index for gastrectomy ^139, 140^, and impaired cellular immunity due to uraemia for haemodialysis ^142^. Nonetheless, several studies among haemodialysis patients have suggested increased rates of CMV (re)infection, either or not associated with transfusion of blood or blood products ^143– 147^, as well as increased rates of CMV reactivation ^143^.

***Solid organ transplantation.*** The incidence of symptomatic CMV infection is strongly increased in solid organ transplant patients, mainly due to infection from a CMV IgG positive donor ^148^. Solid organ transplantation also increases the risk of TB disease ^141, 149– 151^. TB incidence is highest in lung transplant patients and associated with presence of latent TB infection, clinical condition and intensity of the immunosuppressive therapy; the latter has been brought forward as the sole explanation for the increased TB risk ^152^. Interestingly, a study among Korean solid organ transplant patients found that the risk of developing TB was associated with CMV infection within the prior 3 months ^153^.

## Potential impact on tuberculosis control and elimination

If indeed CMV (re-)infection or reactivation commonly precipitates progression from latent infection to active TB, this will suggest novel approaches to TB control. Combining a test for Mtb infection with one for ongoing or recent active CMV infection may strongly increase our ability to predict the development of TB disease and allow the targeting of preventive treatment to those most at risk ^4^. Vaccination against CMV might prevent TB in those with TB infection. A wide range of CMV vaccines are currently in clinical development including plasmid-based vaccines, viral vector vaccines, attenuated HCMV strains, and recombinant protein and peptide vaccines ^154^. Recently, a genetically modified CMV vector expressing antigens from Mtb (RhCMV/TB) has shown some protection against Mtb in a non-human primate study ^155^. If the human version of this vaccine was able to afford (partial) protection against CMV, it could also significantly impact the TB epidemic. However, there is currently no widely available HCMV vaccine and it is unclear if vaccines based on HCMV in humans will offer similar protection to those based on RhCMV in non-human primates. Since CMV infection may also affect TB treatment response, another potential application therefore could be the provision of CMV antiviral treatment as an adjunct to TB treatment, for example of patients with multidrug resistance. 

## Future research

There is an urgent need for elucidating the role of CMV infection in TB disease progression. Further serological and cellular studies should be done to confirm the association between TB disease and CMV infection. However, in settings with high CMV seroprevalence (also those with highest TB incidence) it will be important to identify recent reinfection for which various diagnostic approaches exist ^30^. Their relative merits are beyond the scope of this article, but some may potentially signal reactivation due to Mtb replication, i.e. consequence rather than cause. Therefore, ultimately longitudinal studies are needed in which the incidence of TB disease among those with latent TB infection is measured over time comparing those with CMV (re-)infection or reactivation to those without. These studies should be supplemented with immunological studies to define the mechanisms through which CMV precipitates progression to active TB disease. Finally, it will be important to study the role of CMV reactivation during TB disease and its effect on the response to TB treatment.

## Data availability

No data is associated with this article.
