# Supplementary material for: The convergent epidemiology of tuberculosis and human cytomegalovirus infection
Source: F1000Res. 2018 Apr 30;7:280. Originally published 2018 Mar 6. [Version 2] doi: 10.12688/f1000research.14184.2 (PMC5934687; doi:10.12688/f1000research.14184.2)
Supplement: Peer review history

# Peer review: Referee response for version 2

**DOI:** 10.5256/f1000research.16087.r33563

**Published:** 2018-05-03

**Authors:** Maziar Divangahi

**Affiliations:** 1 McGill Internation TB Center, University of McGill, Montréal, QC, Canada

The authors effectively addressed all my concerns.

I have read this submission. I believe that I have an appropriate level of expertise to confirm that it is of an acceptable scientific standard.

# Peer review: Referee response for version 1

**DOI:** 10.5256/f1000research.15429.r32121

**Published:** 2018-03-22

**Authors:** Maziar Divangahi

**Affiliations:** 1 McGill Internation TB Center, University of McGill, Montréal, QC, Canada

Our continuous exposure to a multitude of pathogens requires a greater understanding of the host response to heterologous infections and is fundamental for developing novel therapies and vaccines against infectious diseases. In line with this, heterologous immunity to viruses and bacteria is an extremely complex scenario as our immune response greatly differs upon viral versus bacterial infection. Thus, how our immune system makes a balanced response to control both viral and bacterial infections without killing the host is a fascinating area of research.

 In this review article, Cobelens and colleagues reviewed the available epidemiological data for human cytomegalovirus (HCMV) and *Mycobacterium tuberculosis *( *Mtb*) infections. The review is well written and provides interesting human observations that HCMV may contribute to the progression of latent TB to active disease. However, I have three major comments that potentially can improve the conceptual idea of the current review.   

 1) Type I IFN is a chief antiviral cytokine, which also plays a critical role in immunity to CMV infection ( *Plos Pathogens*, 10:e1003962, 2014). However, it has been well documented that type I IFN increases host susceptibility to some bacterial infections including *Francisella tularensis* ( *J. Immunol. *169, 1665-1668, 2002), *Listeria monocytogenes* ( *J. Exp. Med. *185:921, 1997) and *Mtb *( *J. Interferon Cytokine Res. *25:694, 2005 & *Nature,* 511:99, 2014). This concept has been also supported by another recent study (JID, 290:270 2014) demonstrating influenza infection increased susceptibility to Mtb infection in a Type I IFN dependent manner. Although the authors briefly mention this idea, a cohesive expansion of this concept would provide further insight for the important role of type I IFN in viral infection and susceptibility to Mtb ( *Trends in Immunology*, 36:307, 2015).     

 2) The authors need to be cautious about their conclusions regarding a recent paper using nonhuman primate (rhesus macaques) cytomegalovirus vectors encoding Mtb antigens (RhCMV/MTB) ( *Nature Medicine*, 24:130, 2018) for the vaccine in TB. Although the protective data from this study are very impressive, the lack of cellular mechanism (e.g. the protection was independent of effector T cells) as well as the lack of several important controls including the empty vector (RhCMV) indicate that further investigations are required to have a better understanding of this approach as a vaccine in pre-clinical study prior to any human trial for TB.

 3) The presented human observations that sexual contact and whole blood transfusion are the risk factors for tuberculosis is very speculative. Thus, the author should consider rephrasing this section at least in the abstract as it sounds very confirmative.   

 Minor comments:

 1)  The Age-specific death rates from TB in England and Wales was also reported for 1913 and 1918 that shows the same pattern (Table 2) and the authors may want to include this reference:

 Langford, C. The age pattern of mortality in the 1918–1919 influenza pandemic: an attempted explanation based on data for England and Wales. Med. Hist. 46, 1–20 (2002).

 2) As there is no evidence of Mtb activating latent HCMV in the lungs (Page 3), which is also not the focus of this review, the author may want to consider removing this entire paragraph.

     

I have read this submission. I believe that I have an appropriate level of expertise to confirm that it is of an acceptable scientific standard, however I have significant reservations, as outlined above.

# Response

**Published:** 2018-04-22

**Authors:** Frank Cobelens

**Affiliations:** Academic Medical Center of the University of Amsterdam, Netherlands

We thank Dr. Divangahi for his valuable comments.

Here we respond point-by-point, referring to the revised version of our paper.

Major comments

1: "(...) Although the authors briefly mention this idea, a cohesive expansion of this concept would provide further insight for the important role of type I IFN in viral infection and susceptibility to Mtb". 

**Response**: The reviewer is right to point out that Type I IFN plays a role in HCMV infection as well as in Mtb infection. We expanded the paragraph on viral triggers to include more detail on the possible role of Type I IFN.

2. "The authors need to be cautious about their conclusions regarding a recent paper using nonhuman primate (rhesus macaques) cytomegalovirus vectors encoding Mtb antigens (RhCMV/MTB) (Nature Medicine, 24:130, 2018) for the vaccine in TB. (...)"

**Response**: We agree with this criticism and now phrased this more cautiously by pointing out that there is currently no widely available HCMV vaccine and it is unclear if vaccines based on HCMV in humans will offer similar protection to those based on RhCMV in non-human primates.

3. "The presented human observations that sexual contact and whole blood transfusion are the risk factors for tuberculosis is very speculative. Thus, the author should consider rephrasing this section at least in the abstract as it sounds very confirmative."   

**Response**: We agree that for these two risk factors the evidence base is limited, although we do point out reasons why (e.g. the confounding effect between TB and sexual contact by HIV status). We rephrased this statement in the abstract as "(...) including poor socio-economic status, solid organ transplantation and, possibly, sexual contact and whole blood transfusion.".

Minor comments

1. "The age-specific death rates from TB in England and Wales was also reported for 1913 and 1918 that shows the same pattern (Table 2) and the authors may want to include this reference:

Langford, C. The age pattern of mortality in the 1918–1919 influenza pandemic: an attempted explanation based on data for England and Wales. Med. Hist. 46, 1–20 (2002)."

**Response**: Indeed TB mortality showed a peak in 1918 in England and Wales, as it did in Switzerland and South Africa (as referenced), as well as in other countries such as The Netherlands (not referenced). This mortality peak has generally been associated with the Spanish Flu epidemic rather than with WW-I, among other because several of these countries were not involved in the war. We feel that this point is rather secondary to our reasoning and does not need additional referencing. Note that mortality peak in 1913 observed by Langford in in England and Wales was observed only in the age group 0-4 years, with little bearing on overall mortality.

In light of this discussion we feel it is important to point out that increased TB mortality during influenza epidemics may reflect increased case fatality among TB patients due to secondary influenza rather than increased TB incidence. We now added this consideration to the paragraph on viral triggers of TB.

2. "As there is no evidence of Mtb activating latent HCMV in the lungs (Page 3), which is also not the focus of this review, the author may want to consider removing this entire paragraph."

**Response**: We agree that this aspect is outside the focus of our review and took out the part of the sentence related to Mtb infection reactivating HCMV infection. We did leave in the remainder of the paragraph but did include a statement that the effect of HCMV on the immunity to Mtb may be systematic as well as local, i.e. in the lungs.

# Peer review: Referee response for version 1

**DOI:** 10.5256/f1000research.15429.r31552

**Published:** 2018-03-14

**Authors:** Blair L. Strang

**Affiliations:** 1 Institute for Infection and Immunity, St George's, University of London, London, UK

The interaction of different pathogens in an infected individual is a fascinating subject, but, in general, poorly understood. For the most part, it is thought that infection with one pathogen facilities the replication and pathogenesis of another. An example of this is the clear and long standing link between human immunodeficiency virus (HIV) infection and development of severe disease related to MTB mycobacterium tuberculosis (MTB) infection. What is interesting of MTB infection are the concepts of predisposing or preexisting infection other than HIV linked to MTB (for example, the widespread respiratory pathogen influenza.)

 In this article, the authors outline intriguing lines of converging evidence detailing the possible association of human cytomegalovirus (HCMV) and MTB infection, suggesting that HCMV infection is a relevant factor in the incidence of MTB disease.

 The evidence presented rests largely on epidemiological findings involving age distribution and congruent risk factors. There is an interesting link between the incidence of MTB in early life and adolescence that mirrors acquisition of HCMV either during pregnancy/post partum or by sexual contact in adolescence. Several congruent risk factors are discussed. However, the authors concede that these factors could be applicable to many widespread opportunistic pathogens, including HCMV.

 Overall, the argument presented based on epidemiological evidence is compelling and the possible link between HCMV and MTB infection should be investigated further. Indeed, the authors should have done more to highlight very resent work from one of their laboratories on this topic. This study, mentioned in the opening paragraphs, shows a clear association of the presence of T cells recognizing HCMV and an increased risk of MTB disease early in life. What is especially interesting about this study is the possibility that development of MTB disease is related to the impairment of NK cell function. Much recent work in HCMV pathogenesis by Wilkinson and colleagues has highlighted the many and diverse mechanisms that HCMV employs to evade NK cells. It is interesting to consider that HCMV evasion of the immune response to infection is directly related to development of MTB disease.

 Other points surrounding mechanisms linking HCMV and MTB infection are also unclear. The authors mention that the lung may be a reservoir for HCMV. However, this is based on studies in mice using murine cytomegalovirus (MCMV). The pathology of MCMV does not accurately mirror that of HCMV. Thus, more compelling evidence relating the presence of HCMV in the lung with disease must be presented. Based on the data discussed above, co-incident infection of HCMV and MTB in lung tissue may not be required for disease, but, perhaps, modulation of the immune system.

 Finally, the authors consider therapeutic intervention. They highlight the sterling work of Picker and colleagues using rhesus cytomegalovirus (RhCMV) as a vaccine vector. These studies have shown that in non-human primates a RhCMV-MTB vaccine candidate can offer notable protection against challenge with MTB. It must be noted, however, that there is currently no widely available HCMV vaccine and it is unclear if vaccines based on HCMV in humans will offer similar protection to those based on RhCMV in non-human primates. It may be some time before viable HCMV based vaccines are available. In the short term it may be better to peruse the other suggestion the authors make, which is to trial the use of anti-HCMV drugs in patients with MTB.

I have read this submission. I believe that I have an appropriate level of expertise to confirm that it is of an acceptable scientific standard.

# Response

**Published:** 2018-04-22

**Authors:** Frank Cobelens

**Affiliations:** Academic Medical Center of the University of Amsterdam, Netherlands

We thank Dr. Strang for his valuable comments.

Here we respond point-by-point, referring to the revised version of our paper.

1. "(... )Indeed, the authors should have done more to highlight very resent work from one of their laboratories on this topic. This study, mentioned in the opening paragraphs, shows a clear association of the presence of T cells recognizing HCMV and an increased risk of MTB disease early in life. What is especially interesting about this study is the possibility that development of MTB disease is related to the impairment of NK cell function. Much recent work in HCMV pathogenesis by Wilkinson and colleagues has highlighted the many and diverse mechanisms that HCMV employs to evade NK cells. It is interesting to consider that HCMV evasion of the immune response to infection is directly related to development of MTB disease."

**Response**: We agree that the possible role of impairment of NK cell function deserves more attention. We now elaborate on this aspect in the section entitled "Human Cytomegalovirus Infection".

2. "The authors mention that the lung may be a reservoir for HCMV. However, this is based on studies in mice using murine cytomegalovirus (MCMV). The pathology of MCMV does not accurately mirror that of HCMV. Thus, more compelling evidence relating the presence of HCMV in the lung with disease must be presented. Based on the data discussed above, co-incident infection of HCMV and MTB in lung tissue may not be required for disease, but, perhaps, modulation of the immune system."

**Response**: We added the following references: Gordon et al, J Exp Med 2017 (a study of donor organs that showed the lung is a major site for CMV DNA) and Poole et al. J Infect Dis 2015 (that found CMV in alvealoar macrophages). But it is also true that CMV may not need to be present in the lung as it impacts the systemic immune response and therefore has an indirect effect still relevant for lung immunity. It is possible that both co-infection of lung AND CMV impact on systemic immune response contribute to TB risk in the CMV infected individual. We added this consideration to the in the section entitled "Human Cytomegalovirus Infection".

3. "It must be noted, however, that there is currently no widely available HCMV vaccine and it is unclear if vaccines based on HCMV in humans will offer similar protection to those based on RhCMV in non-human primates." 

**Response**: We agree with this caveat that we added to the text.
